# Supplementary material for: The Systems Biology Research Tool: evolvable open-source software
Source: BMC Syst Biol. 2008 Jun 29;2:55. doi: 10.1186/1752-0509-2-55 (PMC2446383; doi:10.1186/1752-0509-2-55)
Supplement: Additional file 1 — SBRT Archive. An archive of the current version of the Systems Biology Research Tool. [file 1752-0509-2-55-S1.zip › sbrt-1.4.0/doc/developers_guide/api/sbrt/shell/package-use.html]

Uses of Package sbrt.shell


|  |  |  |
| --- | --- | --- |
| **Overview** \| **Package** \| Class \| **Use** \| **Tree** \| **Deprecated** \| **Index** \| **Help** | |  |
| PREV   NEXT | **FRAMES**    **NO FRAMES**     **All Classes** |  |


---


## **Uses of Package sbrt.shell**

| Packages that use sbrt.shell | |
| --- | --- |
| **sbrt.shell** | Provides classes used by shell-level software. |
| **sbrt.shell.mng.algebra** | Provides classes for creating algebra process managers. |
| **sbrt.shell.mng.fba** | Provides classes and interfaces for creating Flux Balance Analysis process managers. |
| **sbrt.shell.mng.geom** | Provides classes and for creating geometry process managers. |
| **sbrt.shell.mng.stat** | Provides classes and interfaces for creating statistical process managers. |
| **sbrt.shell.text** | Provides classes and interfaces for defining formats for basic objects. |

Classes in sbrt.shell used by sbrt.shell

****ApplicationException****
  
          This class is the superclass of those exceptions that are
expected to be thrown from the SBRT shell.

Classes in sbrt.shell used by sbrt.shell.mng.algebra

****ApplicationException****
  
          This class is the superclass of those exceptions that are
expected to be thrown from the SBRT shell.

Classes in sbrt.shell used by sbrt.shell.mng.fba

****ApplicationException****
  
          This class is the superclass of those exceptions that are
expected to be thrown from the SBRT shell.

Classes in sbrt.shell used by sbrt.shell.mng.geom

****ApplicationException****
  
          This class is the superclass of those exceptions that are
expected to be thrown from the SBRT shell.

Classes in sbrt.shell used by sbrt.shell.mng.stat

****ApplicationException****
  
          This class is the superclass of those exceptions that are
expected to be thrown from the SBRT shell.

Classes in sbrt.shell used by sbrt.shell.text

****ApplicationException****
  
          This class is the superclass of those exceptions that are
expected to be thrown from the SBRT shell.

---


|  |  |  |
| --- | --- | --- |
| **Overview** \| **Package** \| Class \| **Use** \| **Tree** \| **Deprecated** \| **Index** \| **Help** | |  |
| PREV   NEXT | **FRAMES**    **NO FRAMES**     **All Classes** |  |


---
